# Allelic Variations in Phenology Genes in Club Wheat (*Triticum compactum*) and Their Association with Heading Date

**DOI:** 10.3390/ijms26104875

**Published:** 2025-05-19

**Authors:** Bárbara Mata, Adoración Cabrera

**Affiliations:** Genetics Department, Escuela Técnica Superior de Ingeniería Agronómica y de Montes, Campus de Rabanales, Universidad de Córdoba, CeiA3, 14071 Córdoba, Spain; b62maalb@uco.es

**Keywords:** club wheat, *Triticum compactum*, flowering date, vernalization, photoperiod, genetic variability

## Abstract

The allelic diversity within genes controlling the vernalization requirement (*VRN1*) and photoperiod response (*PPD1*) determines the ability of wheat to adapt to a wide range of environmental conditions and influences grain yield. In this study, allelic variations at the *VRN-A1*, *VRN-B1*, *VRN-D1* and *PPD-D1* genes were studied for 89 accessions of *Triticum compactum* from different eco-geographical regions of the world. The collection was evaluated for heading date in both field and greenhouse experiments under a long photoperiod and without vernalization. Based on heading date characteristics, 52 (58.4%) of the genotypes had a spring growth habit, and all of them carried at least one dominant *VRN1* allele, while 37 (41.6%) accessions had a winter growth habit and carried the triple recessive allele combination. The photoperiod-sensitive *Ppd-D1b* allele was detected in 85 (95.5%) accessions and the insensitive *Ppd-D1a* allele in four (4.5%) accessions. A total of 10 phenology gene profiles (haplotypes) were observed at four major genes in the *T. compactum* germplasm collection. The LSD test revealed significant differences in the mean heading date among the different spring phenology gene profiles, both in greenhouse and field conditions. In addition, 21 microsatellite markers (simple sequence repeats, SSRs) were used to assess the genetic diversity in the collection. The 21 SSR markers amplified a total of 183 alleles across all the genotypes, with a mean of 3.2 alleles per locus. The polymorphic information content ranged from 0.49 to 0.94, with a mean of 0.84. The results of this study may be useful for both *T. compactum* and common wheat breeding programs as a source of agronomic traits.

## 1. Introduction

Club wheat (*Triticum compactum* [*Triticum aestivum* ssp. *compactum* (Host) Mac Key, 2n = 6x = 42, A^u^A^u^BBDD]) is a subspecies of common wheat (*T. aestivum* ssp. *aestivum*, 2n = 6x = 42, A^u^A^u^BBDD) that is distributed throughout the Old World [1]. The main areas for cultivation of club wheat are the U.S. Pacific Northwest and some areas of the Middle East (Afghanistan, Iran, Pakistan), Asia (Armenia, Turkey), Europe (Austria, Switzerland) and Australia [2,3]. It is a free-threshing wheat with rounded grains that differs from common wheat in certain morphological characteristics, including its compact spike due to shorter rachis segments. In addition, it has smaller grains than common wheat, but this smaller size is compensated for by a higher seed number [3]. The dominant *C* locus mapped on the short arm of chromosome 2D determines the compact spike [3,4] and also has a pleiotropic effect on many agronomic traits such as spike length, seed size and seed number, influencing crop yield [3,5]. Club wheat is commercially important for producing flours suitable for making cakes and pastries. Some of its quality characteristics, such as low gluten strength, low water absorption, low batter viscosity and low protein, result in high-quality biscuits and cakes [6,7]. Screening for genes related to agronomic traits in club wheat germplasm has shown variability for high molecular weight glutenin [8,9], waxy proteins [10] and stripe rust resistance [11]. It has also been suggested that club wheat cultivars are better adapted to some agroclimatic regions and may be more competitive in dryland areas than common wheat [2,3,12].

The development of new wheat varieties better adapted to water-limited environments is one of the main challenges for plant breeders. The use of the genetic resources available for wheat is key for obtaining new genotypes adapted to climate change. Species in the primary wheat gene pool, such as *T. compactum*, could be good candidates as gene sources for new allelic variants to enable the diversification of phenological characteristics of common wheat cultivars. The transition to flowering in cereals is mainly determined by temperature and photoperiod. In hexaploid wheat, the vernalization-induced flowering pathway is mainly regulated by the MADS-box transcription factor VERNALIZATION1 (*VRN1*) loci [13,14,15]. Allelic variants at the *VRN-A1*, *VRN-B1* and *VRN-D1* homeoloci map to the long arm of chromosomes 5A, 5B and 5D [16,17,18], respectively, and they are one of the main resources of genetic variation in vernalization requirements in wheat. The dominant *VRN1* alleles conferring a spring growth habit result from insertions and/or deletions in the promoter and intron 1 regions [15,19,20,21]. PHOTOPERIOD (*PPD1*), a member of the pseudo-response regulator (PRR) family, is a key gene determining the sensitivity to day length in wheat [22,23]. *PPD1* loci map to the short arm of chromosomes 2A, 2B and 2D, and among them, *PPD-D1* is considered the most important photoperiod regulator in wheat [23,24]. The semi-dominant *Ppd-D1a* allele, characterized by a large deletion upstream of the coding region, leads to photoperiod insensitivity [25,26].

Studies on the diversity of phenology-related genes in *T. compactum* are scarce. The characterization of wheat germplasm for genes controlling heading date variations will provide a valuable tool for breeders in developing wheat varieties that can adapt to various environmental conditions. This study aimed to use functional molecular markers to evaluate the allelic variation in four major genes controlling phenology (*VRN-A1*, *VRN-B1*, *VRN-D1* and *PPD-D1*) in 89 accessions of hexaploid wheat *T. compactum* and to study the effect of allele combination on heading date. The genetic diversity in the club collection was also estimated.

## 2. Results

### 2.1. Allelic Variation at the VRN1 and PPD-D1 Genes in the T. compactum Collection

**Figure 1:**
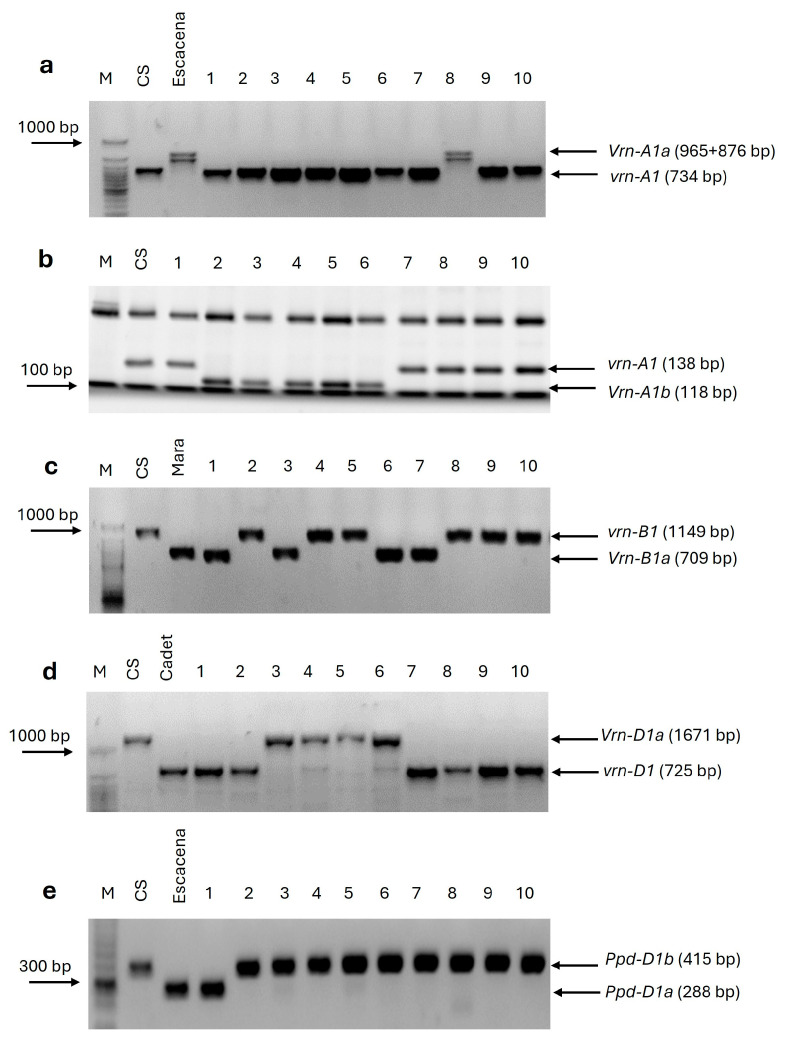
PCR amplification using allele-specific primers for *VRN-A1*, *VRN-B1*, *VRN-D1* and *PPD-D1* loci of different *T. compactum* genotypes. (**a**) Primers VRN1-AF and VRN1-INT1R to detect dominant *Vrn-A1a* and *Vrn-A1b* and recessive *vrn-A1* alleles of the *VRN-A1* gene; (**b**) MspI restriction patterns of the corresponding PCR products in Figure 1a, separated by polyacrylamide gels; (**c**) primers Intr1/B/F, Intr1/B/R3 and Intr1/B/R4 to detect *Vrn-B1a*, *Vrn-B1b* and *vrn-B1* alleles of the *VRN-B1* gene; (**d**) primers Intr1/D/F, Intr1/D/R3 and Intr1/D/R4 to detect *Vrn-D1a*, *Vrn-D1s* and *vrn-D1* alleles of the *VRN-D1* gene; (**e**) primers Ppd-D1-F, Ppd-D1-R1 and Ppd-D1-R2 to detect dominant *Ppd-D1a* photoperiod-insensitive and recessive *Ppd-D1b* photoperiod-sensitive alleles of the *PPD-D1* gene. Common wheat cultivars Chinese Spring (CS), Mara, Cadet and Escacena were used as controls.

Amplification of genomic DNA using specific primers to detect allelic variations in the promoter region of the *VRN-A1* gene allowed the identification of two dominant alleles (*Vrn-A1a* and *Vrn-A1b*) and one recessive allele (*vrn-A1*). PCR products of 965 + 876 bp in length corresponded to the *Vrn-A1a* allele, while those 734 bp in length corresponded to *Vrn-A1b* (714 bp) or *vrn-A1* (734 bp). To differentiate between these latter alleles, the PCR product was digested with the MspI restriction endonuclease, and the fragments were separated using polyacrylamide gels. We obtained two restriction patterns, one 119 bp long corresponding to the dominant *Vrn-A1b* allele and the other, 138 bp long, corresponding to the recessive *vrn-A1* allele. Overall, 18 (20.2%) of the club wheat accessions carried the foldback element insertion characteristic of the *Vrn-A1a* allele, 12 (13.5%) the 119 bp restriction fragment characteristic of the *Vrn-A1b* allele and 59 (66.3%) the 138 bp fragment corresponding to the recessive *vrn-A1* allele (Figure 1a,b).

To study allelic variation at the *VRN-B1* gene, multiplex PCR was performed using three primers (Table S1). In principle, these primers can detect two dominant (*Vrn-B1a* and *Vrn-B1b*) alleles and one recessive (*vrn-B1*) allele [19]. Screening with these primers found two alleles in the club wheat collection studied: the dominant *Vrn-B1a* allele, which was identified in 31 (34.8%) accessions, and the recessive *vrn-B1* allele, which was found in 58 (65.2%) (Figure 1c). The dominant *Vrn-B1b* allele was not detected in the *T. compactum* germplasm evaluated in this study.

To identify allelic variation at the *VRN-D1* gene, multiplex PCR screening was carried out using three primers (Table S1). With these primers, two dominant (*Vrn-D1a* and *Vrn-D1s*) alleles and one recessive (*vrn-D1*) allele can be detected [19,27]. Two alleles were identified in the club wheat collection: the dominant *Vrn-D1a* allele in 11 (12.4%) accessions and the recessive *vrn-D1* allele in 78 (87.6%) (Figure 1d). No *T. compactum* genotypes were found to carry the *Vrn-D1s* allele. In total, 52 (58.4%) accessions carried at least one of the dominant vernalization alleles, and 37 (41.6%) accessions carried recessive alleles at all three *VRN1* vernalization loci.

**Figure 2:**
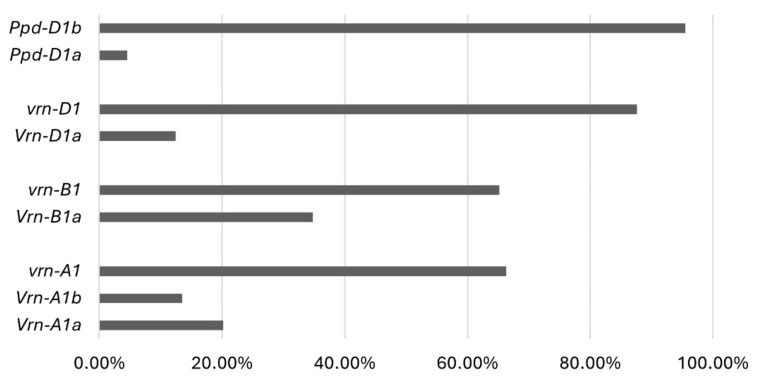
Allele frequencies in the *VRN-A1*, *VRN-B1*, *VRN-D1* and *PPD-D1* genes in the *T. compactum* collection.

Finally, two PCR products (Figure 1e) were obtained from the club wheat collection with the *PPD-D1*-specific primers: the dominant *Ppd-D1a* photoperiod-insensitive and the recessive *Ppd-D1b* photoperiod-sensitive alleles were present in four (4.5%) and 85 (95.5%) accessions, respectively. Figure 2 shows the allele frequencies of the major effect genes, *VRN-A1*, *VRN-B1*, *VRN-D1* and *PPD-D1*, in the *T. compactum* germplasm studied. The allelic variants of vernalization and photoperiod sensitivity genes in each genotype of the club wheat collection studied are listed in Table S2.

### 2.2. Effect of VRN1 and PPD-D1 Allele Combinations on T. compactum Heading Date

Heading date was assessed by growing the club wheat collection under a long photoperiod without vernalization in both the greenhouse and the field. The data gathered was normally distributed under both growth conditions (Figure S1). Out of the 89 *T. compactum* genotypes tested, 37 (41.6%) were classified as having a winter growth habit. These accessions did not flower under either greenhouse or field conditions, and all of them carried recessive (*vrn-A1*-*vrn-B1*-*vrn-D1*) vernalization alleles at all three *VRN1* loci. Table 1 shows the relationship of heading date with vernalization and photoperiod genotypes in the *T. compactum* germplasm under both greenhouse and field conditions.

A total of 10 phenology gene profiles (haplotypes) of four major genes (*VRN-A1*, *VRN-B1*, *VRN-D1* and *PPD-D1*) were observed in the *T. compactum* germplasm collection (Table 1). Only one accession was found to carry the triple dominant *Vrn-A1a*-*Vrn-B1a*-*Vrn-D1a* (1.1%) allele combination at all three loci (haplotype 1). This accession also carried the semi-dominant *Ppd-D1a* allele for photoperiod insensitivity. We also observed combinations of two dominant alleles, namely, *Vrn-A1a*-*Vrn-B1a* (6.7%), *Vrn-A1b*-*Vrn-B1a* (10.1%) and *Vrn-B1a*-*Vrn-D1a* (3.4%). A single dominant allele was observed for *Vrn-A1a* (12.3%), *Vrn-A1b* (3.4%), *Vrn-B1a* (13.5%) and *Vrn-D1a* (7.9%). All these two-dominant and one-dominant-allele combinations carried the recessive *Ppd-D1b* allele for photoperiod sensitivity. The wild-type allele *Ppd-D1b*, causing photoperiod sensitivity, was present in 95.5% of the accessions, whereas only four accessions carried the semi-dominant photoperiod-insensitive *Ppd-D1a* allele (4.5%).

The analysis of variance revealed significant differences (*p* < 0.05) in mean heading date among the different spring allele combinations under both greenhouse and field conditions (Table 1). Under greenhouse conditions, the LSD test found no significant differences in the mean heading date among haplotype 1 (*Vrn-A1a*-*Vrn-B1a*-*Vrn-D1a-Ppd-D1a*), haplotype 2 (*Vrn-A1a*-*Vrn-B1a-vrn-D1-Ppd-D1b*), haplotype 3 (*Vrn-A1b*-*Vrn-B1a-vrn-D1-Ppd-D1b*), haplotype 4 (*vrn-A1*-*Vrn-B1a-Vrn-D1a-Ppd1b*), haplotype 8 (*vrn-A1*-*vrn-B1-Vrn-D1a-Ppd1b*) and the spring common wheat cultivar Escacena (*Vrn-A1a*-*vrn-B1*-*Vrn-D1a-Ppd-D1a*) used as control. The LSD test also showed that the club wheat genotypes carrying haplotype 6 (*Vrn-A1b*-*vrn-B1*-*vrn-D1-Ppd-D1b*) headed significantly later than those harboring triple or double dominant allele combinations at the *VRN1* loci, but no significant differences were found in the mean heading date between haplotypes 6, 5 (*Vrn-A1a*-*vrn-B1*-*vrn-D1-Ppd-D1b*) and 7 (*vrn-A1-Vvrn-B1a*-*vrn-D1-Ppd-D1b*), all of them carrying one dominant allele at the *VRN1* loci. From field data, the LSD test showed that the *T. compactum* accession with haplotype 1 carrying all the dominant alleles at both the *VRN1* and *PPD-D1* loci (*Vrn-A1a*-*Vrn-B1a*-*Vrn-D1a-Ppd-D1a*) and the spring common wheat cultivar Escacena used as a control headed significantly earlier than all the other spring allele combinations. Furthermore, although haplotype 5 (*Vrn-A1a*-*vrn-B1*-*vrn-D1-Ppd-D1b*) carrying a single dominant *Vrn-A1a* allele headed later than haplotype 4 (*vrn-A1*-*Vrn-B1a-Vrn-D1a-Ppd1b*) carrying two dominant alleles and haplotypes 6, 7 and 8 carrying one dominant allele (*Vrn-A1b*, *Vrn-B1a* or *Vrn-D1a*, respectively), the differences were not statistically significant under field conditions.

### 2.3. Geographical Distribution of VRN1 and PPD-D1 Alleles and Allele Combinations

The highest frequencies in the *Vrn-A1a* allele were found in countries from Southeastern Europe (42.9%), whereas *Vrn-A1b* was mainly detected (66.7%) in germplasm from Australia (Table 2). Our results also showed that the *Vrn-A1a* allele was not present in accessions from Africa or Southeast/Central Asia, whereas the *Vrn-A1b* allele was not detected in accessions from Southeastern, Western or Central Europe. The most frequent allele at the *Vrn-A1* locus was the recessive *vrn-A1* allele (66.3%), and it was detected in all geographical areas except Australia. The dominant *Vrn-B1a* allele was present in germplasm from all regions studied, and it showed the highest frequency in accessions from Africa (100%) and Australia (88.9%). The *Vrn-D1a* allele was most frequent in Southeast Asia (69.2%), though it was also detected in genotypes from the USA. Our studies also showed a low frequency (4.5%) of the *Ppd-D1a* allele in the club wheat germplasm evaluated in this study. This allele was only detected in two accessions from the USA, one from Spain and one from Switzerland. By contrast, the photoperiod-sensitive *Ppd-D1b* seems to be much more common and was present at high frequencies in accessions from all geographical regions tested.

**Figure 3:**
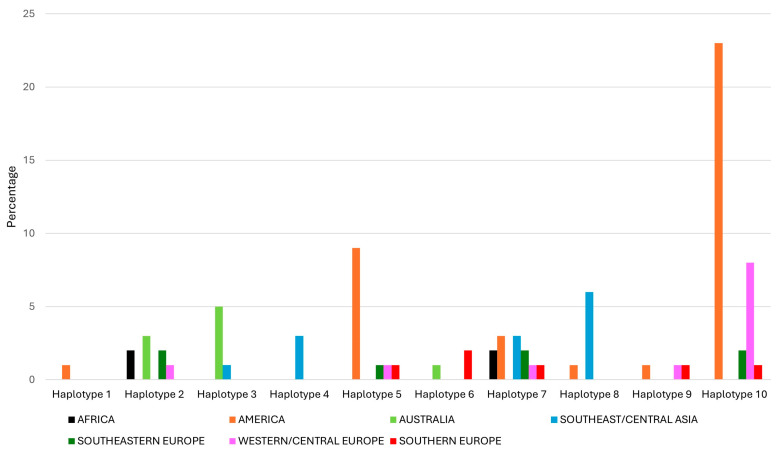
Distribution of the different combinations of vernalization and photoperiod alleles (haplotypes) in a *T. compactum* germplasm collection by geographical origin.

Figure 3 shows the distribution of the ten haplotypes by geographical origin. Haplotype 1 (*Vrn-A1a*-*Vrn-B1a*-*Vrn-D1a*-*Ppd-D1a*) carrying the dominant alleles for the *VRN-A1*, *VRN-B1*, *VRN-D1* and *PPD-D1* genes was detected in the cultivar Calorwa from the USA. Haplotypes 3 and 6 were mainly distributed in Australia, Southeast/Central Asia and Southeastern Europe. Haplotype 4 (*vrn-A1*-*Vrn-B1a*-*Vrn-D1a*-*Ppd-D1b*) only occurred in Southeast/Central Asia, specifically in accessions from Kazakhstan and Kyrgyzstan. Haplotype 7 (*vrn-A1*-*Vrn-B1a*-*vrn-D1*-*Ppd-D1b*) was the most common of the spring genotypes, and it was also the most universally distributed across all continents, except Australia. Haplotype 8 was mainly found in Southeast/Central Asia. Finally, winter genotypes (haplotypes 9 and 10) were common in America and Southern Europe, Western/Central Europe and Southeastern Europe, while they were absent in Africa, Australia and Southeast/Central Asia.

### 2.4. Simple Sequence Repeat Marker and Cluster Analysis

A total of 183 alleles were detected across all 89 club wheat accessions analyzed in this study using 21 simple sequence repeat (SSR) markers (Table 3; Table S3). The allele sizes ranged from 54 (*Xgwm44*) to 280 bp (*Xgwm397*). The mean number of alleles per marker was 3.2, ranging from 1 to 8.9. All the SSR markers, except *Xgwm44* and *Xgwm391*, were considered informative markers (PIC > 0.5). The mean PIC value estimated was 0.84. The phylogenetic relationship among the *T. compactum* accessions was investigated by cluster analysis.

**Figure 4:**
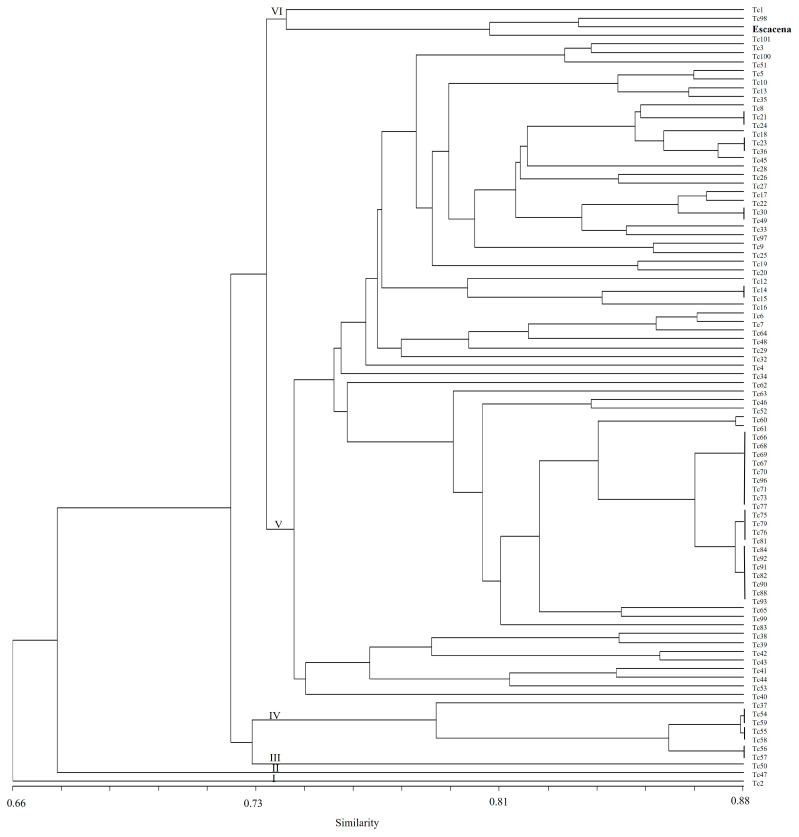
Unweighted pair group method with arithmetic mean dendrogram obtained from cluster analysis of 89 *T. compactum* accessions based on the Dice similarity coefficient using 21 SSR markers.

The dendrogram indicates six main groups (Figure 4). The first group is separated from all the others and contains one accession (Tc2), a landrace from Kyrgyzstan. Groups II and III also contain just one accession each (Tc47 and Tc50, respectively), both from the USA. Group IV consists of seven accessions (T37, T54, T55, T56, T57, T58 and T59), all of them landraces from Pakistan. Group V was divided into two subgroups: Group V-1 is composed of eight accessions, five of them from Australia (Tc41, Tc42, Tc43, Tc,44; Tc53), two from Africa (Tc39, Tc40) and one from Pakistan (Tc38), and Group V-2 is a wider cluster containing 68 accessions from all eco-geographical regions studied except Africa. Finally, Group VI comprises three accessions (Tc1, Tc98 and Tc101), one collected in Mexico, one in (the then) Czechoslovakia and one in Georgia. The common wheat cultivar Escacena was also included in this cluster.

Clusters I, IV, V-1 and VI contained accessions with a spring growth habit (haplotypes 1 to 8) and clusters II and III accessions with a winter growth habit (haplotypes 9 and 10), while cluster V-2 contained both spring and winter growth habit accessions.

## 3. Discussion

The use of diagnostic DNA markers has allowed us to establish the allelic composition of *VRN1* and *PPD-D1* genes affecting the vernalization requirement and photoperiod response, respectively, in a *T. compactum* germplasm collection representing a wide range of eco-geographical regions. Within the screened germplasm, the highest allele frequency (87.6%) was recorded for the recessive *vrn-D1* allele, followed by the recessive *vrn-A1* (66.3%) and *vrn-B1* (65.2%) alleles. The photoperiod-sensitive *Ppd-D1b* allele was detected in 85 (95.5%) accessions and the insensitive *Ppd-D1a* allele in four (4.5%). The *Ppd-D1a* allele was found in accessions from Spain, Switzerland and the USA. In common wheat, the *Ppd-D1a* allele has been mostly found in Central and Southern European [26] and Asian [28,29,30] varieties, whereas the photoperiod-sensitive *Ppd-D1b* allele was most common among wheat varieties from the USA and Canada [28].

Among the genotypes with at least one dominant *VRN1* allele, the frequencies of the different alleles varied across geographical regions (Table 2, Figure 3). The *Vrn-A1a* allele has been found to be the most frequent dominant allele in common spring wheat cultivars in many parts of the world [15,21,31,32]. In this study, the *Vrn-B1a* allele was the most frequent dominant allele in spring *T. compactum* accessions evaluated, with a frequency of 34.8%, followed by *Vrn-A1a* (20.2%), *Vrn-A1b* (13.5%) and *Vrn-D1a* (12.4%). In common wheat, the *Vrn-A1b* allele was found in commercial cultivars from Southern Europe [33] and landraces from Turkey [34]; in the *T. compactum* germplasm collection evaluated in this study, this allele occurred in all regions tested except Southeastern Europe and Western/Central Europe, being most frequent in Australia. The *Vrn-A1b* allele was also found in some genotypes of club wheat from Afghanistan [35].

We did not find the dominant *Vrn-B1b* allele in any of the *T. compactum* accessions studied, consistent with previous reports [35,36]. Dragovich et al. [35] found that 66.7% of *T. compactum* accessions contained haplotype 7 (*vrn-A1-Vrn-B1a-vrn-D1-Ppd-D1b*); they considered this haplotype to be species-specific and also suggested that this allelic composition was characteristic of Asian club wheats. In accordance with their results [35], in the collection we studied, haplotype 7 was the most frequent (13.5%) among the *T. compactum* accessions with a spring growth habit. On the other hand, we also found that haplotype 7 was the most widely distributed, occurring in all regions tested except Australia. In this study, we found that haplotype 4 (*vrn-A1-Vrn-B1a-Vrn-D1a-Ppd-D1b*) was the only allelic combination found in Southeast/Central Asia (Figure 3), although it was also present in genotypes from Africa, Bulgaria, the UK and the USA.

Three alleles (*Vrn-D1a*, *Vrn-D1s* and *vrn-D1*) were detected at the *VRN-D1* gene in a multiplex reaction with the three primers used (Table S1). In the *T. compactum* germplasm analyzed here, the dominant *Vrn-D1a* allele and the recessive *vrn-D1* allele were identified. The *Vrn-D1s* allele, caused by DNA transposon insertion in intron 1, was identified in *T. aestivum* ssp. *spelta* [27]. The same authors also found this allele in one accession of *T. compactum* and concluded that it is not specific to hexaploid *T. spelta*. In this study, we did not find the *Vrn-D1s* allele in any of the accessions evaluated. On the other hand, this allele was the only dominant allelic variant at the *VRN-D1* locus found in a Spanish spelt collection, supporting the hypothesis that *Vrn-D1s* is specific to *T. spelta* [37].

Based on the heading date characteristics of the club wheat accessions assessed, 52 (58.4%) of the genotypes had a spring seasonal growth habit and 37 (41.6%) a winter growth habit. Accordingly, analysis with gene-specific molecular markers confirmed the presence of the recessive alleles at all three *VRN1* loci in winter genotypes, whereas at least one dominant *VRN1* allele was present in spring genotypes. The LSD test revealed significant differences in mean heading date among the spring *T. compactum* genotypes, showing that the allele combinations at *VRN1* influenced seed head emergence under both greenhouse and field conditions, as previously reported in common wheat [38,39].

The mean heading date did not differ significantly between the local spring wheat cultivar Escacena (*Vrn-A1a-vrn-B1-Vrn-D1a-Ppd-D1a*) and the *T. compactum* cultivar Calorwa carrying the dominant (*Vrn-A1a*-*Vrn-B1a*-*Vrn-D1a-Ppd-D1a*) combination for both *VRN1* and *PPD-D1* alleles (haplotype 1) under greenhouse or field conditions. All haplotypes headed significantly later than Calorwa under field conditions; however, this cultivar was the only *T. compactum* accession carrying the dominant alleles for both *VRN1* and *PPD-D1* genes. Therefore, we cannot rule out the possibility that the significant LSD data are not statistically representative due to the small sample size. In wheat, alleles other than those studied here have been found to influence heading date [27,40,41,42] as well as copy number variations in *VRN1* alleles [43,44,45], and hence, we cannot rule out such alleles affecting the phenology of *T. compactum* accessions.

Genetic diversity in the *T. compactum* genotypes was assessed by capillary electrophoresis using 21 genomic SSR markers covering the A, B and D genomes. All the primers used in this study were designed for common wheat, and all of them successfully amplified products in *T. compactum*. This result is consistent with that previously found by Zhang et al. [46], who investigated the transferability of sequence-tagged SSR markers from common wheat to 18 related grass species and found full transferability (100%) for *T. compactum*, supporting the genetic proximity of these subspecies. The dendrogram obtained in this study did not separate the *T. compactum* accessions from the common wheat cultivar Escacena. Specifically, this cultivar clustered in the same group as accessions Tc1, Tc98 and Tc101, providing evidence for the genetic proximity of club wheat and common wheat germplasm [46].

**Figure 5:**
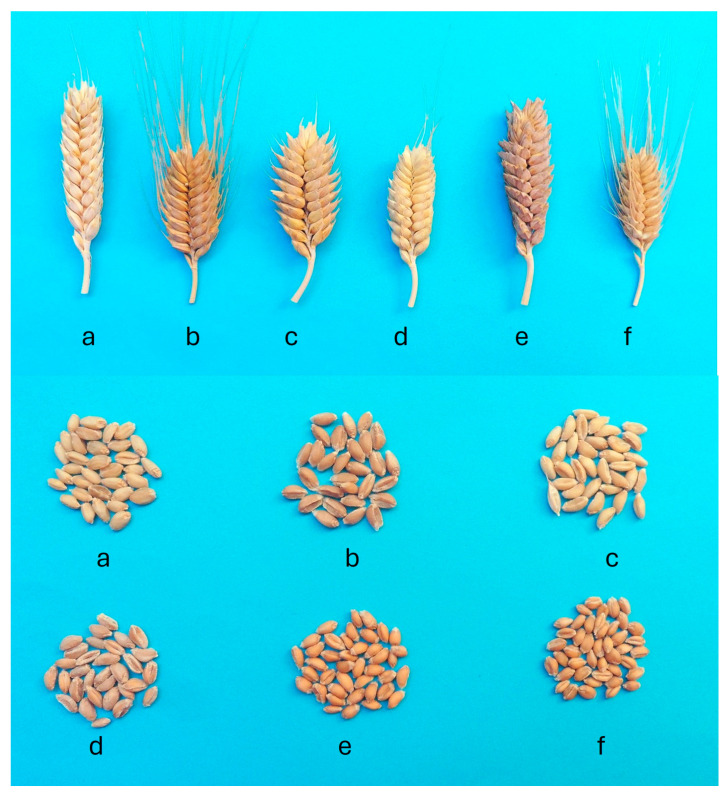
Spike and seed morphology of different *T. compactum* accessions. From left to right: (**a**) PI 25970; (**b**) PI 60740; (**c**) PI 164160; (**d**) PI 191542; (**e**) PI 294567 and (**f**) PI 330540.

A total of 183 alleles were detected from 21 SSR markers among the 89 *T. compactum* genotypes. The *Xgwm565* molecular marker was the most informative SSR marker, with a PIC value of 0.92, identifying 21 alleles (Table 3). The mean number of different alleles was 8.7, with a mean number of alleles per locus of 3.2. The mean number of alleles per locus obtained in different germplasm collections of common wheat varies from 2.5 [47,48] and 3.1 [46] to 5.05 [49], 5.4 [50] or 6.5 [51] alleles per locus. The mean PIC value calculated in *T. compactum* in this study was 0.84, which is higher than the values of 0.4 [46], 0.43 [50] and 0.69 [48] obtained with SSRs in common wheat in previous studies. Both the PIC value and the mean number of alleles per locus revealed a high level of genetic diversity in the *T. compactum* germplasm collection. The *T. compactum* accessions included in this study were from 24 different countries. This broad geographic distribution of the club wheat accessions in the collection may explain the high diversity observed. This diversity is also seen in spike and seed morphology (Figure 5).

On the other hand, we found accessions with identical SSR patterns that are clustered together in the dendrogram, and they also share both haplotype and geographical origin. For instance, Tc56 and Tc57 are both from Pakistan, and Tc14 and Tc15 are from Kazakhstan. These findings suggest that certain accessions might be duplicated and that SSR studies can aid in detecting duplicate accessions.

## 4. Materials and Methods

### 4.1. Plant Material

Eighty-nine accessions of *T. compactum* obtained from the National Small Grains Collection (Aberdeen, ID, USA) were employed in this study (Table S2). The collection included landraces, cultivars and breeding materials from 23 countries across the world. Spring wheat cultivars, namely, Chinese Spring, Cadet, Escacena and Mara, were used as controls.

### 4.2. Phenotyping

Greenhouse and field experiments were carried out in the 2023–2024 growing season in Córdoba, in southern Spain. For the greenhouse experiments, two germinated seeds for each accession were sown individually in plastic pots and grown at 20–25 °C under long-day conditions (18 h day) without vernalization. The heading date was measured in days from emergence until 50% of the spike had emerged from the flag leaf on the main stem, and the mean was calculated from the two plants grown per accession.

For evaluating the ear emergence time of club wheat accessions in the field, the seeds were sown on 1 March 2024, that is, under long-day conditions, in a randomized complete block design with two replications without vernalization. Each experimental unit was a single-row plot (50 cm long) containing at least 20 plants, with a 35 cm space between adjacent plots. The heading date was measured in 50% of the plants in the plot, and as in the greenhouse experiments, by counting the number of days from emergence until 50% of the spike had emerged from the flag leaf.

### 4.3. Genotyping

#### PCR Amplification of Allele-Specific Markers

Total genomic DNA was isolated from frozen young (four-leaf stage) plants using the cetyltrimethylammonium bromide method [52]. To prepare DNA samples for each accession, equal amounts of leaves from three individual plants of each accession were bulked. The samples were stored at −20 °C until amplification through PCR. The concentration of DNA in each sample was estimated using a NanoDrop 1000 Spectrophotometer (Thermo Scientific, Waltham, MA, USA).

Genotypes were characterized for the *VRN1* (*VRN-A1*, *VRN-B1* and *VRN-D1*) and *PPD-D1* genetic loci using a set of functional molecular markers. Dominant (*Vrn-A1a* and *Vrn-A1b*) and recessive (*vrn-A1*) alleles of the *VRN-A1* locus were detected using the primers VRN1AF and VRN1-INTR1R, as described by Yan et al. [15]. To distinguish between *Vrn-A1b* and *vrn-A1*, the PCR product was digested with restriction endonuclease MspI as in Shcherban et al. [33], and DNA fragments were separated on polyacrylamide gels. Allelic variants of the *VRN-B1* gene were identified using three gene-specific primers (Intr1/B/F, Intr1/B/R3 and Intr1/B/R4), a combination linked to intron 1 in a multiplex PCR reaction, which allows detection of *Vrn-B1a*, *Vrn-B1b* and *vrn-B1* [12,19]. Primers Intr1 and Intr1/B/R3 were used to differentiate between *Vrn-B1a* and *Vrn-B1c* [33].

To detect *VRN-D1* intron 1 alleles, a multiplex PCR was performed using three primers (Intr1/D/F, Intr1/D/R3 and Intr1/D/R4). This PCR makes it possible to distinguish between *Vrn-D1a*, *Vrn-D1s* and *vrn-D1* allelic variants [19,27]. Further, three specific primers (Ppd-D1-F1, Ppd-D1-R1 and Ppd-D1-R2) were used in a multiplex PCR assay to identify both the photoperiod-insensitive allele *Ppd-D1a* and photoperiod-sensitive allele *Ppd-D1b* of the *PPD-D1* gene, respectively [25]. The primer sequences used in this study and annealing temperatures for each primer combination are listed in Table S1. PCR was performed with 40 ng of template DNA in a 25 µL volume reaction mixture containing 5 µL of 1× PCR buffer, 0.4 µM of each primer, 1.5 mM of MgCl_2_, 0.4 mM of dNTPs and 0.25U of Taq DNA polymerase (Promega, Madison, WI, USA). The PCR conditions were as follows: 4 min at 94 °C, followed by 35 cycles of denaturation at 94 °C for 45 s, annealing for 45 s, extension at 72 °C for 1 min and then a final extension at 72 °C for 5 min. Amplified products were resolved on 2.0% agarose gels or separated by polyacrylamide gels (10% *w*/*v*, C: 2.67%) and stained with Safe View Nucleic Acid Stain (NBS Biologicals, Huntingdon, UK) incorporated in the gel. Amplicon lengths were determined using Kodak Digital Science 1D software (version 2.0).

### 4.4. Simple Sequence Repeat Analysis

To evaluate the genetic variation in 89 accessions of *T. compactum*, we selected 21 primers developed in wheat by Röder et al. [53]. The commercial common wheat variety Escacena was included in the analysis. The PCR amplification (final volume: 10 µL) consisted of 20 ng of genomic DNA, 2 mM of MgCl_2_, 0.8 mM of dNTP, 5× polymerase buffer, 0.125 µM of forward primer, 0.25 µM of reverse primer, 0.25 µM of fluorochromes (FAM or 6-HEX) and 1 U of Taq polymerase (Promega). The forward primers were synthesized with a 19 bp long M13 tail (5′-CACGACGTTGTAAAACGAC-3′). The cycling protocol was: 1 min at 94 °C, 35 cycles at 94 °C for 1 min, annealing at 53 °C for 1 min and polymerization at 72 °C for 1.5 min, followed by a final extension at 72 °C for 10 min. The amplification products were separated using an automated capillary sequencer (ABI 3130 Genetic Analyzer, Applied Biosystems/HITACHI, Madrid, Spain) in the Genomics Unit of the Central Research Support Service at the University of Córdoba. The size of the amplified bands was determined based on an internal DNA standard (400HDROX) with GeneScan software (version 3.x), and the results were interpreted using Genotyper software (Version 3.7), both from Applied Biosystems. The number of different alleles and polymorphic information content (PIC) were also determined [54]. The Dice coefficient was calculated as a measure of similarity between accessions. Cluster analysis was performed using the unweighted pair group method with arithmetic means, a hierarchic agglomerative method equivalent to the average linkage between groups method. A dendrogram was constructed using the Numerical Taxonomy and Multivariate Analysis System software (NTSYS Version 2.0, Applied Biostatistics, Setauket, NJ, USA).

### 4.5. Statistical Analysis

To evaluate the statistical significance of differences in heading date between different allele combinations for *VRN1* and *PPD-D1* loci in the club wheat collection, an analysis of the variance was conducted, and the means were compared with the least significant difference (LSD) test (*p* < 0.05) using the Statistix software package, version 8.0. The local common wheat cultivar Escacena was included as a control.

## 5. Conclusions

The knowledge of diversity in major phenology genes may be useful in *T. compactum* breeding programs as a source of agronomically valuable traits. Furthermore, the club wheat germplasm evaluated in this study could provide new genetic resources needed for developing wheat varieties adapted to new agroecological conditions.

## Figures and Tables

**Table 1:** Mean number of days ± SE from sowing to heading for *VRN1* and *PPD-D1* allele combinations in the *T. compactum* collection studied.

Haplotype	Allelic Composition				No. of Accessions (%)	Average Heading Time (No. of Days to Heading ± SE)	
						Greenhouse	Field
-	^1^ ***Vrn-A1a***	*vrn-B1*	***Vrn-D1a***	***Ppd-D1a***	-	40.0 ± 0.5 a	62.5 ± 1.5 a
1	***Vrn-A1a***	***Vrn-B1a***	***Vrn-D1a***	***Ppd-D1a***	1 (1.1%)	41.0 ± 0.7 a	61.0 ± 0.1 a
2	***Vrn-A1a***	***Vrn-B1a***	*vrn-D1*	*Ppd-D1b*	6 (6.7%)	40.3 ± 4.1 a	75.9 ± 2.5 bc
3	***Vrn-A1b***	***Vrn-B1a***	*vrn-D1*	*Ppd-D1b*	9 (10.1%)	54.5 ± 3.3 ab	75.0 ± 2.6 bc
4	*vrn-A1*	***Vrn-B1a***	***Vrn-D1a***	*Ppd-D1b*	3 (3.4%)	54.7 ± 4.8 ab	82.0 ± 3.5 cde
5	***Vrn-A1a***	*vrn-B1*	*vrn-D1*	*Ppd-D1b*	11 (12.3%)	61.9 ± 3.0 bc	84.8 ± 1.8 e
6	***Vrn-A1b***	*vrn-B1*	*vrn-D1*	*Ppd-D1b*	3 (3.4%)	66.3 ± 5.7 c	80.0 ± 1.7 cde
7	*vrn-A1*	***Vrn-B1a***	*vrn-D1*	*Ppd-D1b*	12 (13.5%)	60.2 ± 2.9 bc	80.7 ± 1.7 cde
8	*vrn-A1*	*vrn-B1*	***Vrn-D1a***	*Ppd-D1b*	7 (7.9%)	50.3 ± 3.8 ab	82.5 ± 2.3 cde
9	*vrn-A1*	*vrn-B1*	*vrn-D1*	***Ppd-D1a***	3 (3.4%)	No spikes	No spikes
10	*vrn-A1*	*vrn-B1*	*vrn-D1*	*Ppd-D1b*	34 (38.2%)	No spikes	No spikes

^1^ Bread wheat Escacena cultivar used as control. Means with the same letters in the same column are not significantly different for an LSD test at *p* < 0.05. Dominant alleles are in bold.

**Table 2:** Frequencies (%) of the allelic variants of the *VRN-A1*, *VRN-B1*, *VRN-D1* and *PPD-D1* genes in a *T. compactum* germplasm collection by geographical origin.

	*VRN-A1*			*VRN-B1*		*VRN-D1*		*PPD-D1*	
	*Vrn-A1a*	*Vrn-A1b*	*vrn-A1*	*Vrn-B1a*	*vrn-B1*	*Vrn-D1a*	*vrn-D1*	*Ppd-D1a*	*Ppd-D1b*
Africa (4) ^a^	0	2 (50) ^b^	2 (50)	4 (100)	0	0	4 (100)	0	4 (100)
America (38)	9 (23.7)	1 (2.6)	28 (73.7)	5 (13.1)	33 (86.9)	2 (5.3)	36 (94.7)	2 (5.3)	36 (94.7)
Australia (9)	3 (33.3)	6 (66.7)	0	8 (88.9)	1 (11.1)	0	9 (100)	0	9 (100)
Southeast/Central Asia (13)	0	1 (7.7)	12 (92.3)	7 (53.8)	6 (46.2)	9 (69.2)	4 (30.8)	0	13 (100)
Southeastern Europe (7)	3 (42.9)	0	4 (57.1)	4 (57.1)	3 (42.9)	0	7 (100)	0	7 (100)
Western/Central Europe (12)	2 (16.7)	0	10 (83.3)	2 (16.7)	10 (83.3)	0	12 (100)	1 (16.7)	11 (83.3)
Southern Europe (6)	1 (16.7)	2 (33.3)	3 (50.0)	1 (16.7)	5 (83.3)	0	6 (100)	1 (16.7)	5 (83.3)
Total (89)	18 (20.2)	12 (13.5)	59 (66.3)	31 (34.8)	58 (65.2)	11 (12.4)	78 (87.6)	4 (4.5)	85 (95.5)

^a^ Number of accessions, ^b^ percentage.

**Table 3:** Marker size range, number of alleles and polymorphic information content (PIC) observed in 89 accessions of *T. compactum* studied with 21 SSR markers, the first time they are cited.

Simple Sequence Repeat Marker	Size Range (bp)	Number of Alleles		PIC ^c^
		N ^a^	Mean ^b^	
*Xgwm43*	118–256	7	5.9	0.84
*Xgwm44*	54–58	2	1.0	0.49
*Xgwm55*	111–245	7	5.9	0.83
*Xgwm135*	133–158	7	2.5	0.68
*Xgwm205*	134–184	11	3.2	0.77
*Xgwm219*	79–100	10	1.0	0.70
*Xgwm249*	152–200	6	3.0	0.70
*Xgwm257*	182–214	7	3.1	0.75
*Xgwm271*	172–217	7	3.2	0.94
*Xgwm285*	108–260	19	6.1	0.87
*Xgwm291*	114–138	10	2.8	0.75
*Xgwm294*	83–151	9	1.0	0.75
*Xgwm314*	100–223	8	3.4	0.75
*Xgwm382*	104–216	18	3.7	0.85
*Xgwm391*	134	1	1	-
*Xgwm397*	183–280	3	1.7	0.61
*Xgwm403*	105–129	2	1.6	0.52
*Xgwm513*	150–168	10	3.9	0.80
*Xgwm544*	185–240	7	1.8	0.70
*Xgwm565*	93–246	21	8.9	0.92
*Xgwm614*	131–180	11	2.0	0.77
Total	-	183	-	-
Mean	-	8.7	3.2	0.84

^a^ Number of different alleles. ^b^ Mean number of alleles per accession. ^c^ Calculated using the polymorphic markers.

## Data Availability

Data is contained within the article and Supplementary Materials.

## Supplementary Materials

The following supporting information can be downloaded at: https://www.mdpi.com/article/10.3390/ijms26104875/s1.
