# Supplementary material for: Allelic Variations in Phenology Genes in Club Wheat (Triticum compactum) and Their Association with Heading Date
Source: Int J Mol Sci. 2025 May 19;26(10):4875. doi: 10.3390/ijms26104875 (PMC12112085; doi:10.3390/ijms26104875)
Supplement: Supplementary file 1 [file ijms-26-04875-s001.zip › Table S1 Primers sequences annealing temperatures VRN1 and PPD-D1 modified.pdf]

Mata, B., Cabrera, A. Allelic variations in phenology genes in club wheat (*Tritticum compactum*) and their associations with heading date

Table S1. Primer sequences, annealing temperatures and expected PCR product sizes used for identifying major vernalization (*VRN1*) and photoperiod (*PPD-D1*) alleles in the *T. compactum* collection.

Locus	Primer name	Primer sequence (5′ → 3′)	Annealing temperature	Allelic variant	Expected product size	References
VRN-A1	VRN1-AF<br>VRN1-INT1R	GAAAGGAAAAATTCTGCTCG<br>GCAGGAAATCGAAATCGAAG	60ºC	*Vrn-A1a*<br>*Vrn-A1b*<br>*vrn-A1*	980 bp + 867 bp<br>714 bp<br>734 bp	Yan et al. [1];<br>Shcherban et al. [2]
VRN-A1	Intr1/C/F<br>Inrt1/AB/R	GCACTCCTAACCCACTAACC<br>TCATCCATCATCAAGGCAAA	58ºC	*vrn-A1*	1068 bp	Yan et al. [1];<br>Fu et al. [3]
VRN-B1	Intr1/B/F<br>Intr1/B/R3<br>Intr1/B/R4	CAAGTGGAACGGTTAGGACA<br>CTCATGCCAAAAATTGAAGATGA<br>CAAATGAAAAGGAATGAGAGCA	63ºC	*Vrn-B1a*<br>*Vrn-B1b*<br>*vrn-B1*	709 bp<br>673 bp<br>1149 bp	Fu et al. [3];<br>Milec et al. [4]
VRN-B1	Intr1<br>Intr1/B/R3	ATCATCTTCTCCACCAAGGG<br>CTCATGCCAAAAATTGAAGATGA	58ºC	*Vrn-B1a*<br>*Vrn-B1c*	1124 bp<br>737 bp	Shcherban et al. [2]
VRN-D1	Intr1/D/F<br>Intr1/D/R3<br>Intr1/D/R4	GTTGTCTGCCTCATCAAATCC<br>GGTCACTGGTGGTCTGTGC<br>AAATGAAAAGGAACGAGAGCG	65ºC	*Vrn-D1a*<br>*Vrn-D1s*<br>*vrn-D1*	1671 bp<br>1800 bp<br>997 bp	Fu et al. [3];<br>Muterko et al. [5]
VRN-D1	Intr1/D/F<br>INSD-R	GTTGTCTGCCTCATCAAATCC<br>GCGAGTAGGACGATGTCGAG	60ºC	*Vrn-D1s*	795 bp	Palomino and Cabrera [6]
PPD-D1	Ppd-D1_F<br>Ppd-D1_R1<br>Ppd-D1_-R2	ACGCCTCCCACTACACTG<br>GTTGGTTCAAACAGAGAGC<br>CACTGGTGGTAGCTGAGATT	54ºC	*Ppd-D1a*<br>*Ppd-D1b*	288 bp<br>414 bp	Beales et al. [7]

1.Yan, Helguera, M.; Kato, K.; Fukuyama, S.; Sherman, J.; Dubcovsky, J. Allelic Variation at the *VRN-1* promoter region in polyploid wheat. *Theor. Appl. Genet.***2004**, *109*, 1677–1686. https://doi.org/10.1007/s00122-004-1796-4.

2. Shcherban, A.B.; Börner, A.; Salina, A. Effect of *VRN-1* and *PPD-1* genes on heading time in European bread wheat cultivars. *Plant Breed*. **2015**, *134*, 49–55. https://doi.org/10.1111/pbr.12223.

3. Fu, L.; Szücs, S.P.; Yan, L.L.; Helguera, M.; Skinner, J.S.; Von Zitzewitz, J.; Hayes, P.M.; Dubcovsky, J. Large deletions within the first intron in *VRN-1* are associated with spring growth habit in barley and wheat. *Mol. Genet. Genom.* **2005**, *273*, 54–65. https://doi.org/10.1007/s00438-004-1095-4.

4. Milec, Z.; Tomkova, L.; Sumikova, T.; Pankova, K. A new multiplex PCR test for the determination of *Vrn-B1* alleles in bread wheat (*Triticum aestivum* L.). *Mol Breed*. **2012**, *30*, 317–323. https://doi.org/10.1007/s11032-011-9621-7.

5. Muterko, A.; Balashova, I.; Cockram, J.; Kalendar, R.; Sivolap, Y. The new wheat vernalization response allele *Vrn-D1s* is caused by DNA transposon insertion in the first intron. *Plant Mol. Biol. Repor*. **2015**, *33*, 294–303. https://doi.org/10.1007/s11105-014.

6. Palomino, C.; Cabrera, A. Evaluation of the allelic variations in vernalisation (*VRN1*) and photoperiod (*PPD1*) genes and genetic diversity in a Spanish spelt wheat collection. *Int. J. Mol. Sci*. **2023**, 24, 16041. https://doi.org/10.3390/ijms242216041

7. Beales, J.; Turner, A.; Griffiths, S.; Snape, J.; Laurie, D.A. A pseudo-response regulator is misexpressed in the photoperiod insensitive *Ppd-D1a* mutant of wheat (*Triticum aestivum* L.). *Theor. Appl. Genet.* **2007**, *115*, 721–733. https://doi.org/10.1007/s00122-007-0603-4.
