# Supplementary material for: Allelic Variations in Phenology Genes in Club Wheat (Triticum compactum) and Their Association with Heading Date
Source: Int J Mol Sci. 2025 May 19;26(10):4875. doi: 10.3390/ijms26104875 (PMC12112085; doi:10.3390/ijms26104875)
Supplement: Supplementary file 1 [file ijms-26-04875-s001.zip › Figure S1 Distribution of Tcompactum genotypes by heading date.pdf]

Mata, B., Cabrera, A. Allelic variations in phenology genes in club wheat (*Tritticum compactum*) and their associations with heading date

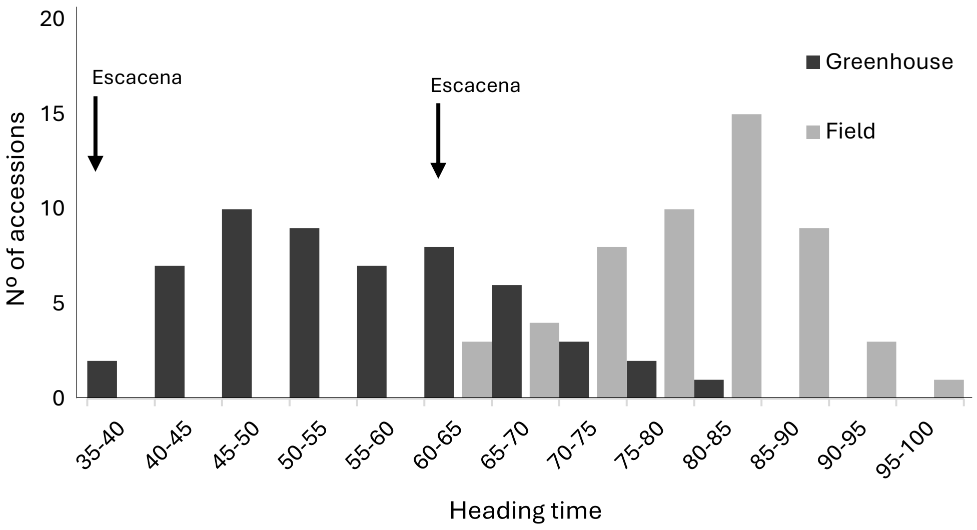

Figure S1. Distribution of *T. compactum* genotypes by heading date under greenhouse (black columns) conditions and field (grey columns) conditions.
